# Genomic Analysis of Reproductive Trait Divergence in Duroc and Yorkshire Pigs: A Comparison of Mixed Models and Selective Sweep Detection

**DOI:** 10.3390/vetsci12070657

**Published:** 2025-07-11

**Authors:** Changyi Chen, Yu He, Juan Ke, Xiaoran Zhang, Junwen Fei, Boxing Sun, Hao Sun, Chunyan Bai

**Affiliations:** College of Animal Science, Jilin University, Changchun 130062, China; forlccy@163.com (C.C.); heyu21@mails.jlu.edu.cn (Y.H.); kejuan23@mails.jlu.edu.cn (J.K.); zhangxr1504@163.com (X.Z.); feijw22@mails.jlu.edu.cn (J.F.)

**Keywords:** additive effect, dominant effect, *F_ST_*, population analysis, reproduction traits

## Abstract

This study aimed to investigate population genetic differences related to reproductive traits between Duroc and Yorkshire (Dutch Large White) pigs using two approaches: linear mixed models that dissect additive and dominant effects, and selective sweep analysis. (1) Methods: Genome-wide single-nucleotide polymorphism (SNP) data of 3917 Duroc and 3217 Yorkshire pigs were analyzed. The first principal component (PC1) was used as a simulated phenotype to capture population-level variance. Additive and dominant genetic effects were partitioned and evaluated by using the combination of the linear mixed models (LMM) and ADDO’s algorithm (LMM + ADDO). In parallel, selective sweep signals were detected using fixation index (*F_ST_*) and nucleotide diversity (θ_π_) analyses. A comparative assessment was then conducted between the LMM + ADDO and the selective sweep analysis results. Significant loci were annotated using quantitative trait loci (QTL) databases and the Ensembl genome browser. (2) Results: There are 39040 SNPs retained after quality control. Using the LMM + ADDO framework with PC1 as a simulated phenotype, a total of 632 significant SNPs and 184 candidate genes were identified. Notably, 587 SNPs and 171 genes were uniquely detected by the LMM + ADDO method and not among loci detected by the top 5% of *F_ST_* and θ_π_ values. Key candidate genes associated with litter size included *HSPG2*, *KAT6B*, *SAMD8*, and *LRMDA*, while *DLGAP1*, *MYOM1*, and *VTI1A* were associated with teat number traits. (3) Conclusions: This study demonstrates the power of integrating additive and dominant effect modeling with population genetics approaches for the detection of genomic regions under selection. The findings provide novel insights into the genetic architecture of reproductive traits in pigs and have practical implications for understanding the inheritance of complex traits.

## Simple Summary

This study investigated genetic differences related to reproductive traits in Duroc and Yorkshire (Dutch Large White) pigs using two approaches: linear mixed models (LMM) with additive and dominant effects, and selective sweep analysis. The first principal component (PC1) was used as a simulated phenotype to capture population variance. A comparative analysis revealed that 587 SNPs and 171 genes were uniquely identified by the LMM + ADDO framework, not overlapping with the top 5% *F_ST_* and the top and bottom 5% of θ_π_ values. Some key genes like *HSPG2*, *KAT6B,* and *SAMD8* were linked to litter size, while *DLGAP1* and *MYOM1* were associated with teat number traits. This integration of additive/dominant modeling and population genetics provided novel insights into the genetic architecture of reproductive traits in pigs, highlighting the value of complementary approaches for understanding complex trait inheritance.

## 1. Introduction

Genomic selection signatures across populations reflect the effects of natural and artificial selection pressures. Cross-population genome-wide comparisons have proven effective in detecting the genetic architecture underlying phenotypic variation. Studies have identified population-specific selection signatures in pigs accounting for adaptive differentiation and trait-specific genetic variation through whole-genome comparisons, such as between Landrace and Yorkshire breeds [1] and Meishan subpopulations [2].

The fixation index (*F_ST_*) is a widely used statistic for identifying selection signatures across populations, quantifying allele frequency divergence. *F_ST_* captures only frequency differences, which correspond to additive effects. However, other genetic effects, e.g., dominance, are important components of the genetic architecture of complex traits, especially those with low heritability, such as reproductive traits in mammals [3]. Linear mixed models (LMMs), as employed in genome-wide association studies (GWAS), allow decomposition of these effects [4] but require individual-level phenotypic data, which are often unavailable in selection signature analyses.

To overcome this limitation, we adopted an EigenGWAS-inspired framework using the first principal component (PC1) of genome-wide SNP variation as a surrogate phenotype. This method, initially proposed by Cortés et al. (2016) [5] and further refined in subsequent studies [6], enables the dissection of genetic effects in the absence of phenotypic data. We further integrated the ADDO (Additive-Dominance Decomposition Orthogonalization) algorithm to efficiently distinguish and visualize additive and dominant contributions to trait variation [6].

We applied this approach to SNP data from two reproductively divergent pig breeds, Duroc and Yorkshire, which serve as typical sire and dam lines in commercial breeding, respectively. Notably, Yorkshire pigs demonstrate significantly higher reproductive output—averaging 35.41 lifetime offspring born alive, compared to 24.83 in Duroc [7]—making them an ideal model for investigating the genetic basis of reproductive performance.

## 2. Materials and Methods

### 2.1. Populations and Genotypes

Dataset 1 comprised SNP chip genotypes of 3917 Duroc pigs with 40,535 SNP loci, obtained from Ruan et al. [8]. Dataset 2 included 3217 Dutch Large White pigs (a breed of Yorkshire) with 47,314 SNP loci, sourced from Deng et al. [9]. Variant call format (VCF) files from both datasets were merged using custom scripts. Standard quality control (QC) steps were applied to the merged data: samples with less than 90% SNP call rate were excluded; missing genotypes were imputed using Beagle v5.0 [10]; SNPs with a minor allele frequency (MAF) below 0.05 were removed.

### 2.2. Dissection of Additive and Dominant Genetic Effects

To capture genetic variance components related to fecundity, we performed a genome-wide dissection of additive and dominance effects. Principal component analysis (PCA) was conducted using GCTA [11]. Because PC1 explained 65.4% of the variance between the two populations, while PC2 accounted for only 19.6%, the first principal component (PC1) was extracted as a surrogate phenotype.

The GWAS was conducted using linear mixed models (LMMs) implemented in the HIBLUP platform [12]. The model is formulated as follows:

$$y=Xb+\sum_{i=1}^{k}Z_{i}u_{i}+e$$

where y represents the vector of phenotypic observations (PC1 values in this study), X is the *n* by $n_{b}$ design matrix for fixed effects and covariates, b is the vector of their corresponding coefficients, and k denotes the total number of genetic random effects. $Z_{i}$ is the n by $n_{i}$ design matrix for the $i_{th}$ genetic random effect and $u_{i}$ is the vector of its corresponding genetic effects with a length of $n_{i}$, which follows the multivariate normality of $N(0,K_{i}\sigma_{i}^{2})$, and $k_{i}$ can be the additive or dominant matrix that is derived from genomic information. The residual term e is the vector of model residuals with a zero mean and a variance of $\sigma_{e}^{2}$. And the Kinship matrices for each population were calculated by HIBLUP.

Furthermore, following the ADDO algorithm [6], we applied constrained least squares optimization to decompose SNP effects into additive and dominance components using singular value decomposition (SVD). Linear regressions were performed on the genomic data in R (v4.4.1) under three models: a null model (excluding genotype effects), an additive model (A model), and a combined additive-dominance model (AD model). Analyses of variance (ANOVA) were conducted to evaluate the significance of SNP effects within the LMM + ADDO framework. The genome-wide significance threshold was set at *p* = 1.3 × 10^−6^, based on a Bonferroni correction (0.05/n, n means the total number of SNPs).

### 2.3. Selection Sweep Detection

To assess genetic differentiation between Duroc and Yorkshire pigs based on allele frequency differentiation, pairwise *F_ST_* values were calculated at each SNP site using vcftools (v1.16.0) [13]. In addition, nucleotide diversity (θπ) and the ratio between populations were computed for each population using PLINK (v1.9) [14] (www.cog-genomics.org/plink/1.9/, accessed on 22 October 2024), providing complementary evidence for selection signals.

### 2.4. Functional Annotation of Significant Loci

For functional annotation of significant loci, we focus on the loci (1) co-identified by different models; (2) detected by dominant effects but not by sweep selection analysis; and (3) subjected to co-selection by both populations by sweep selection analysis. The position relative to functional genes of each SNP, e.g., upstream, downstream, coding region, introns, or intergenic region, was annotated using VEP [15]. Due to the large number of identified loci and genes, we chose to focus mainly on those co-identified loci) for the annotation of genes and the subsequent discussion of the analysis The possible functions of candidate genes were also analyzed by querying major databases (NCBI, Ensembl [16], iswine [17], PigBiobank [18], pig QTLdb [19], etc.) and published studies.

In addition, to verify whether the selected SNPs have effects on proliferation traits in real pig populations, we performed association studies of genotypes with litter size traits in a Dongliao pig population with phenotype records, which has been used in our previous study [20]. The Dongliao pig population contains genotype and Total Born Number (TBN) and Number Born Alive (NBA) phenotype of 723 sows with records of 2499 litters. We performed a multifactor ANOVA to assess the effects of genotype and other environmental factors by R4.4.1 [21].

## 3. Results

### 3.1. Additive and Dominant Effect Detection

Following quality control, 38,040 SNPs were retained from the Duroc and Yorkshire pig populations. Principal component analysis (PCA) was performed using these SNPs. The first two components (PC1 and PC2) accounted for a combined 85.4% of the total genetic variance, with PC1 explaining 65.4% and PC2 explaining 19.6%. Clear population stratification was observed along PC1, with Duroc and Yorkshire pigs forming distinct clusters (Supplementary Materials Figure S1). PC1 was thus used as the phenotypic surrogate for downstream analyses.

**Figure 1:**
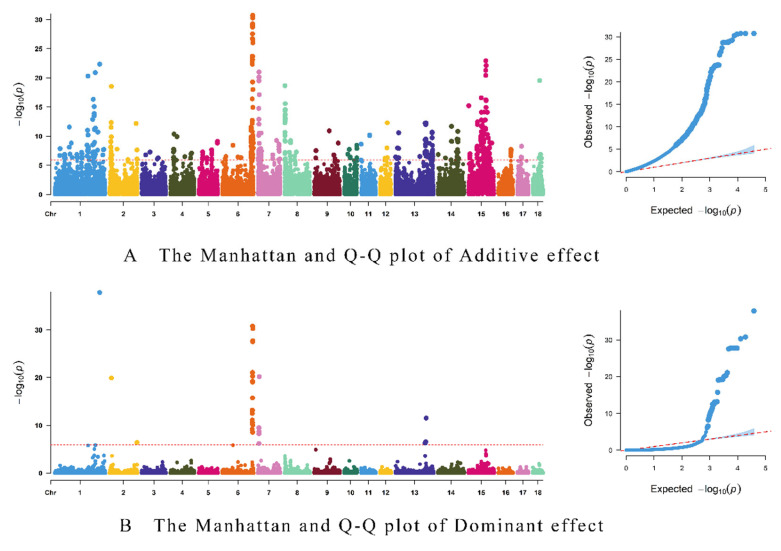
Manhattan and Q-Q plots for additive effects (**A**) and dominant effects (**B**).

Three linear regressions were fitted, with the null model (no genotype effects), the A model (which includes additive genetic effects), and the AD model (which includes both additive and dominant effects), respectively. And 630 additive loci and 47 dominant loci were identified at the level of $p\leq 1.3\times 10^{-6}$ (0.05/n, n denotes the number of SNPs). Notably, 45 out of the 47 dominant loci were also among the additive ones. Manhattan and Q–Q plots of the results are shown in Figure 1, while Figure 1A displays additive effects and Figure 1B dominant effects. The share of additive variance was 75.91% and the share of dominant effect variance was 24.09%. And there are two significant SNPs that were exclusively uniquely identified through dominant effects alone.

### 3.2. The Result of Selection Sweep Detection

**Figure 2:**
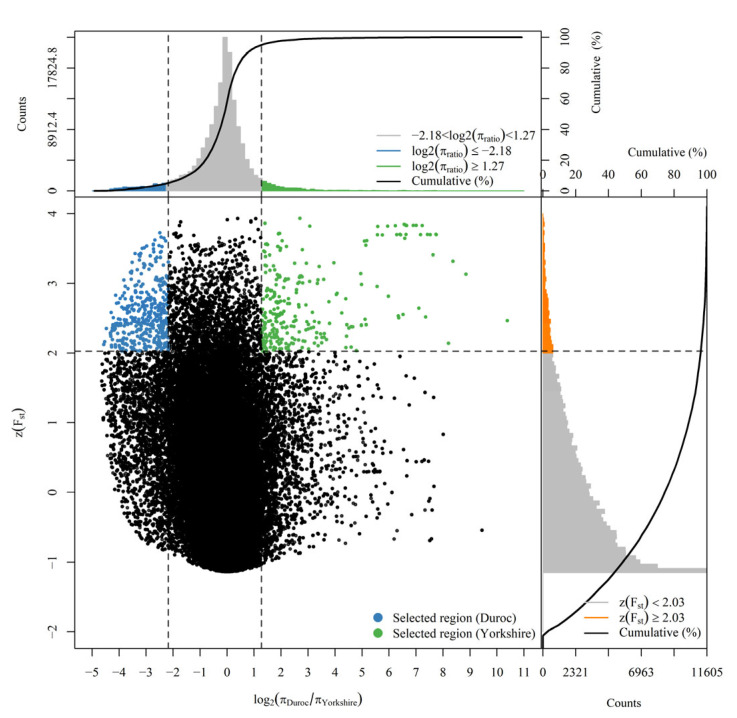
Selective sweep analysis plot for Duroc and Yorkshire. The curves represent the stacking percentages of the bar charts. The blue dots in the center are the Duroc group selected region, and the green dots are the Yorkshire group selected region. The orange area on the right is the highest 5% *F_ST_*.

Candidate loci under selection were identified using a composite approach combining *F_ST_* and nucleotide diversity (θπ) metrics. The Z-score normalization (z(*F_ST_*)) on the values of *F_ST_* was computed by using the mean and standard deviation. The π_ratio_ metric was calculated as the log2- transformation ratio of θπ between two populations $\left(log2\;\left(\pi_{ratio}\right)\right)$, specifically defined as ${log}_{2}\frac{\theta \pi \_Duroc}{\theta \pi \_Yorkshire}$. The genomic regions with both top 5% z(*F_ST_*) and $log2\;(\pi_{ratio})\;$(absolute) values were determined as the selected regions in the two populations, as shown in Figure 2. This screening approach identified 844 loci under selection, with 496 in Duroc and 348 in Yorkshire pigs. The detail information of the results is provided in Supplementary Table S1.

### 3.3. Comparison of LMM + ADDO Framework with F_ST_ Analysis

**Figure 3:**
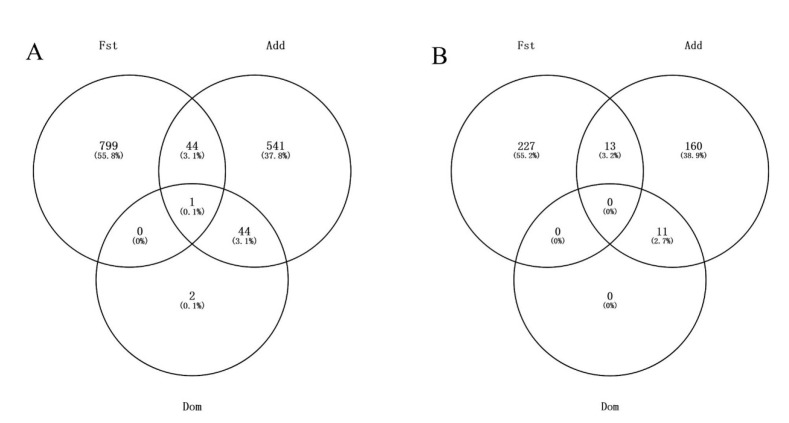
Veen diagrams of SNPs (**A**) and genes (**B**) identified by $F_{st}$, additive effect (Add), and dominant effect (Dom).

A total of 44 loci were shared between the selective sweep and additive effect categories, while only one SNP was common across all three categories: selective sweep, additive effect, and dominant effect (Figure 3A). These findings suggest that the selective sweep analysis method, which is based on allele frequency differentiation, primarily captures additive genetic effects. However, it fails to detect the majority of additive loci and completely overlooks loci with dominant effects. The $F_{st}$ values, along with the *p*-values for additive and dominant effects across all loci, are provided in Supplementary Materials Table S2.

### 3.4. Functional Annotation Results of Significant Loci

For the 630 significant SNPs with significant additive effect, a total of 20 loci identified in this study overlapped with known reproduction trait QTLs (Table S2). A total of 173 genes were annotated by Ensembl for loci with only significant additive effects, while 11 were for those with both significant additive and dominant effects (Figure 3B).

Of the 844 loci identified by selection sweep detection, 29 overlapped with known reproduction QTLs. A total of 170 genes were annotated by Ensembl for loci under selection in the Duroc population, and 135 for those in the Yorkshire population. Among them, nine genes were annotated by those loci under selection in both populations: *HSPG2*, *DLGAP1*, *MYOM1*, *GALNTL6*, *KAT6B*, *SAMD8*, *LRMDA*, *PRKG1*, and *VTI1A*. The detail information of VEP annotation results of each SNP, including variant types such as the non-coding transcript exon variant, intergenic variant, intron variant, and regulatory region variant, is shown in Supplementary Materials Table S3. Some variants in enhancer regions were annotated, such as ENSSSCR1_BMTR5 (chr1:90098790), ENSSSCR6_CLGC9 (chr6:165850427), etc., but their specific functions and effects on porcine reproductive traits need to be further investigated.

**Figure 4:**
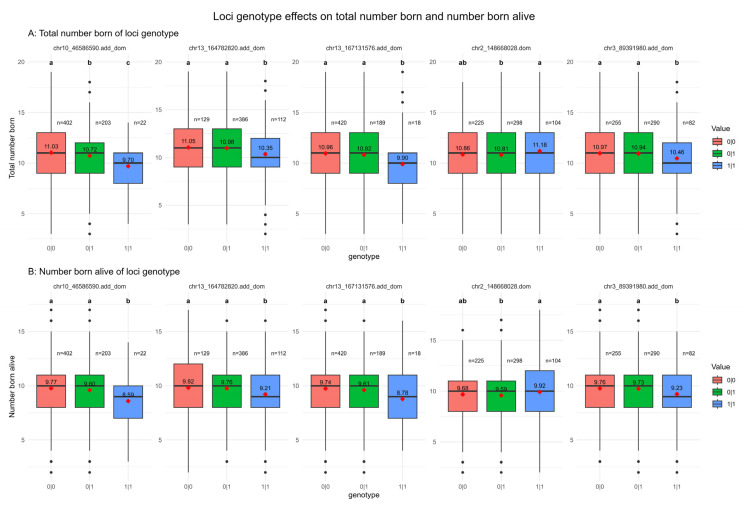
Loci genotype effects on total number born (**A**) and number born alive (**B**). The dom means the locus has a significant dominant effect, add_dom means this locus has a significant additive and dominant effect, 0|0 means no mutant purebred, 0|1 means heterozygous, and 1|1 means mutant purebred. Different letters in the top indicate significant differences (*p* < 0.05) between genotypes. The values in the boxes mean the average of TNB and NBA for individuals of different genotypes, and the n above the boxes represents the sample size.

Figure 4 presents the genotype–phenotype association results for litter size traits in Dongliao black pigs. Due to differences in genotyping platforms between the Dongliao black pig dataset and our study, association analyses were restricted to the five loci shared by both datasets. The locus located on chromosome 2 (chr2:148668028) is the locus with only a significant dominant effect (dom), and the other four loci are the loci that are significant in both additive and dominant effects (add_dom), but not in sweep selection analysis. Heterozygotes (0|1) at locus chr2:148669028 had lower average TNB and NBA compared to the homozygotes (0|0 and 1|1), indicating a potential dominant deleterious effect on litter size. At the other four loci, homozygous (1|1) individuals showed significantly reduced TNB and NBA compared to both heterozygous (0|1) and wild-type homozygous (0|0) genotypes, demonstrating combined additive and dominant genetic effects.

Notably, our LMM + ADDO framework identified some known reproduction traits related to QTLs, which were not identified by selection sweep detection (Table 1).

## 4. Discussion

We hypothesize that dominant genetic effects may play a significant role in shaping the phenotypes of traits with low heritability, such as reproduction characteristics. When aiming to detect dominant effects between populations in genome-wide selection signature analysis, the markers identified as having dominant effects are primarily loci where one population predominantly exhibits heterozygous genotypes, while the other population predominantly exhibits homozygous genotypes. These heterozygous loci can often also be detected by additive and *F_ST_* analyses, though their significance is typically lower compared to loci with distinct homozygote genotypes in different populations. Therefore, when exploring genetic selection signatures between populations, there is a risk of overlooking these heterozygous loci, which may nonetheless play a crucial role in understanding the genetic basis of dominant effects and heterosis. Extensive evidence supports the importance of incorporating dominant effects in genomic selection. Studies have demonstrated that dominant models improve prediction accuracy for crossbreeding performance using purebred data [28,29] and outperform additive models [30]. Specifically, Sun et al. [31] reported superior prediction for dairy yield traits when combining additive and dominant effects compared to additive-only models. Dominance variance accounts for approximately 13% of total genetic variance for litter size in crossbred populations [32], with dominant effects explaining 11–20% of variance for total number born (TNB) compared to just 2–11% for backfat thickness [33]. These findings collectively highlight the necessity of including dominant effects in pig breeding programs to optimize genetic gain.

To further investigate this, we collected genome-wide SNP data from two commercial pig breeds: Duroc and Yorkshire pigs. In commercial pig breeds, the Yorkshire pig breed has a better performance in reproduction traits than the Duroc pig breed. Yorkshire pigs are known for their superior reproduction performance compared to Duroc pigs, which is why they are typically used as dam lines in pig production, whereas Duroc pigs are often utilized as sire lines. The PCA revealed clear genetic differentiation between the two breeds. Thus, using the PC1 as the phenotype, we investigated both additive and dominant effects through the LMM + ADDO framework to explore the genetic basis of reproduction traits. In this study, a total of 632 significant SNPs and 184 genes were identified. Compared with the *F_ST_* analysis, 587 SNPs and 171 genes were newly detected. The *F_ST_* analysis revealed loci selected by Duroc and Yorkshire pigs separately. The analysis results in Duroc and Yorkshire populations demonstrated that both additive and dominant QTLs were identified by LMM + ADDO, with superior performance compared to the *F_ST_* method. Furthermore, we observed that while the *F_ST_* method is effective in detecting additive loci, it performs poorly in identifying dominant effects.

Among these genes, nine co-selected genes by Duroc and Yorkshire pigs were identified, namely *HSPG2*, *DLGAP1*, *MYOM1*, *GALNTL6*, *KAT6B*, *SAMD8*, *LRMDA*, *PRKG1*, and *VTI1A*. A query in the PigBiobank database [18] showed that *HSPG2* [34], *KAT6B, SAMD8*, and *LRMDA* were associated with litter size traits in pigs, while *DLGAP1* [35], *MYOM1*, *GALNTL6*, *PRKG1*, and *VTI1A* exhibited strong correlations with teat size traits. Additionally, *HSPG2* regulates morphogenesis and tissue differentiation, influencing birth weight and growth [36] while also affecting follicular quality and fertility in dairy cattle [37]. Notably, human studies indicate an inverse relationship between *HSPG2* expression and oocyte quality in normal ovulatory cycles [38]. The *GALNTL6* plays a role in male fertility through spermatogenesis regulation in Holstein cattle [39].

Our integrated analysis identified 13 candidate genes (*MYO6*, *OTX2*, *BNC2*, *CCDC171*, *PTPRD*, *LSM1*, *AGPS*, *PDE11A*, *OSBPL6*, *ITGA4*, *FAM117B*, *SORCS2*, and *THSD7A*) showing significant signals in both *F_ST_* and additive effect analyses. Notably, 11 of these genes (excluding *LSM1* and *PDE11A*) are documented in PigBiobank as associated with reproductive performance traits. Functional characterization reveals these genes’ possible roles in proliferation: *OTX2* regulates embryonic development through epigenetic mechanisms [40,41], with its elevated expression correlating with improved fertilization rates in the avian model [42]. *BNC2*, expressed in ovarian somatic cells, is essential for normal ovarian morphology and fertility [43]. *PDE11A*, a known litter size candidate in sheep [44], modulates cAMP hydrolysis critical for gonadotropin signaling [45], *ITGA4* influences both gestation length [46] and nuclear transfer embryo development in pigs [47], while *SORCS2* participates in lipid metabolism [48]. *THSD7A* emerges as a key player in high-altitude adaptation across species [49,50,51].

Importantly, we prioritized loci that were not identified by sweep selection analysis but emerged through our LMM + ADDO framework, underscoring their potential role in porcine reproductive traits. Among the genes annotated, 11 genes—including *DPYSL3*, *TRABD2B*, *CMPK1*, *STIL*, *FAAH*, *MAST2*, *PRDX1*, *TESK2*, and *BTBD19*—were found to be associated with the teat numbers, while *LRRC41* was associated with the litter size. *CMPK1* has been identified across various bovine populations [52], and its association with epithelial ovarian cancer (EOC) has been reported in humans, and knockdown of *CMPK* affects apoptosis in EOC cells [53].

**Figure 5:**
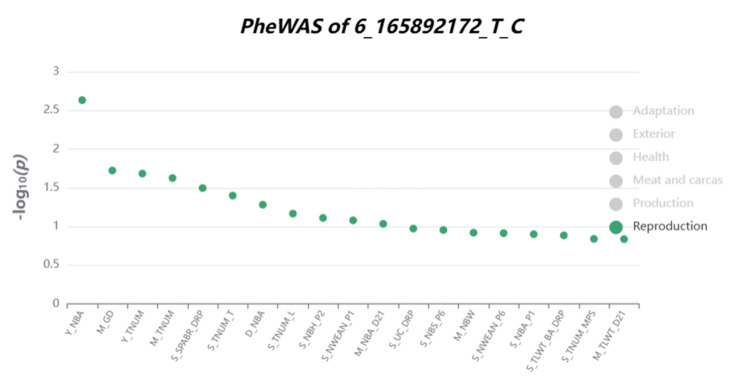
PheWAS results for locus chr6:165892172 (T > C) in PigBiobank. The *y*-axis shows the $-{log}_{10}\;(p-value),$ where higher values indicate greater statistical significance of the association between the locus and each trait. The *x*-axis represents reproduction-related traits. NBA: number born alive; GD: gestation days; TNUM_T: total teat number; DPR: sperm abnormality rate; NBH_P2: number of healthy piglets born at parity 2.

Notably, the loci of chr6:165892172 (T > C) located on *TESK2* were associated with the number of pigs born alive in PigBiobank (Figure 5). Although studies on *TESK2* in pigs are limited, Yang et al. [54] reported its involvement in the early growth and development of calves, and another study linked it to sperm function and fertility [55].

The functions of some candidate loci related to litter size in pigs were further validated through a genotype–phenotype association analysis in an independent pig population with both phenotypic and genotypic data. These findings collectively demonstrate the enhanced precision achieved by combining *F_ST_* analysis with the LMM + ADDO framework to jointly consider both additive and dominant effects. This study also has some limitations since the study was conducted using PC1 as the phenotypic value. More experiments and studies are needed to validate these loci and genes we identified.

## 5. Conclusions

In the realm of genome-wide selection characterization, investigating additive and dominant effects across populations can enable more precise identification of candidate genes, which serve as valuable targets for breeding enhancement. As an additional result, we identified loci and genes potentially associated with porcine reproduction traits. The insights gained from this research deepen our understanding of the genetic mechanisms underlying phenotypic variation in Duroc and Yorkshire pigs. Moreover, our analytical mindset provides a viable method for exploring genome-wide trait selection within populations, offering new perspectives for future studies.

## Figures and Tables

**Table 1:** Noteworthy SNP loci recorded in the QTL database.

Loci	Additive (*p*)	Dominant (*p*)	$F_{st}$	Reproduction QTL (QTL ID)	References
16_156647853	5.24 × 10^−7^	0.720151	0.096352	Gestation length (261,966)	Wang et al., 2017 [22]
7_81948941	1.64 × 10^−7^	0.843502	0.19554	Teat number (37,446)	Duijvesteijn et al., 2014 [23]
8_4451319	2.58 × 10^−15^	0.00373	0.004865	Teat number (223,271)	Bovo et al., 2021 [24]
10_32822679	3.64 × 10^−7^	0.424028	0.258834	Nonfunctional nipples (124,209)	Chalkias et al., 2017 [25]
14_107377294	1.52 × 10^−11^	0.005087	0.002753	Litter size (130,290)	He et al., 2017 [26]
15_75063953	3.91 × 10^−9^	0.049719	0.516495	Number of stillborn (57,518)	Schneider et al., 2015 [27]
16_68542670	1.92 × 10^−8^	0.803131	0.378008	Teat number (223,295)	Bovo et al., 2021 [24]

## Data Availability

All data have been included in the main text and Supplementary Materials.

## Supplementary Materials

The following supporting information can be downloaded at https://www.mdpi.com/article/10.3390/vetsci12070657/s1, Figure S1: PCA; Table S1: Results of *Fst* analysis; Table S2: The *Fst* value and *p*-value of additive and dominant; Table S3: The annotation results of VEP.
