# Supplementary figures and images for: Genomic Analysis of Reproductive Trait Divergence in Duroc and Yorkshire Pigs: A Comparison of Mixed Models and Selective Sweep Detection
Source: Vet Sci. 2025 Jul 11;12(7):657. doi: 10.3390/vetsci12070657 (PMC12299411; doi:10.3390/vetsci12070657)

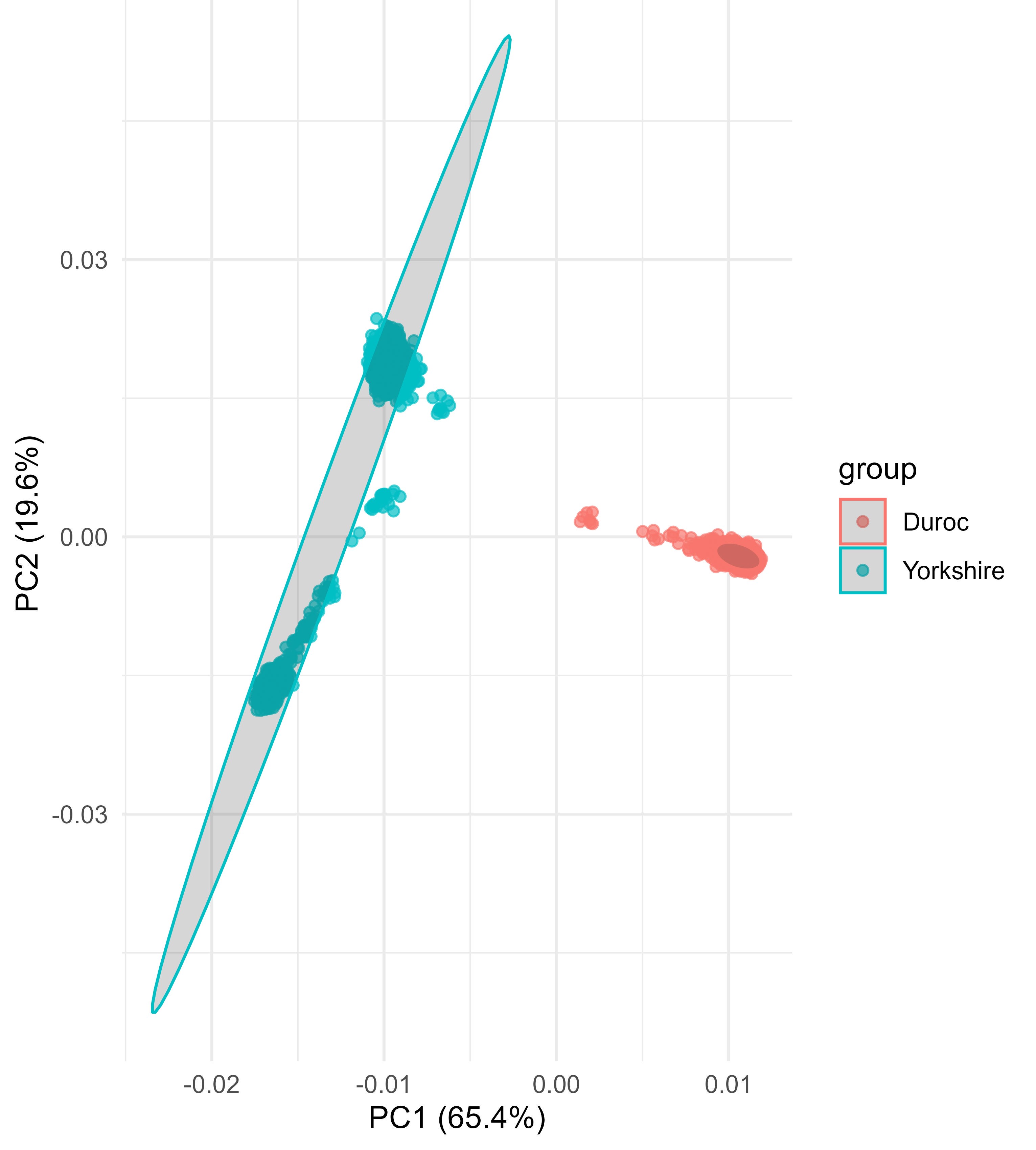

Supplement: Supplementary file 1 [file vetsci-12-00657-s001.zip › Figure S1 PCA.jpg]
